# Drug shops for stronger health systems: learning from initiatives in six LMICs

**DOI:** 10.1186/s40545-021-00374-z

**Published:** 2021-11-16

**Authors:** Geetanjali Lamba, Zubin Cyrus Shroff, Zaheer-Ud-Din Babar, Abdul Ghaffar

**Affiliations:** 1 Alliance for Health Policy and Systems Research, World Health Organization, Geneva, Switzerland; 2 Centre for Pharmaceutical Policy and Practice Research, Department of Pharmacy, University of Huddersfield, Queensgate, Huddersfield, UK

## Abstract

**Background:**

Private sector retail pharmacies, or drug shops, play an important role in access to essential medicines and services in low-and-middle-income countries. Recognising that they have the potential to contribute to health system strengthening efforts, many recent initiatives to engage with drug shops have been launched. These include initiatives that focus on changes in policy, regulation and training. However, the specific factors that influence their success remain poorly understood. Seven country case studies supported under the Alliance’s programme of work ‘Strengthening health systems: the role of drug shops’ help to explore this issue.

**Methods:**

Country case studies from the above programme of research from Bangladesh, Indonesia, Myanmar, Nigeria, Tanzania and Zambia were used as the main sources of data for this paper. A modified version of Bigdeli et al.’s Access to Medicines framework was applied within a partially grounded approach to analyze each country case study and compare themes between countries.

**Results:**

Many factors may help initiatives targeting drug shops successfully achieve their intended outcomes. At the micro level, these include community demand for drug shops and a positive relationship between drug shops and their clients. At the meso level, facilitators of initiative success include training and positive attitudes from drug shops towards the initiative. Barriers include client pressure, procurement challenges and financial and administrative costs associated with initiatives. At the macro level, collaboration between stakeholders, high-level buy in and supervision, monitoring and regulation may influence initiative success. These factors are inter-dependent and interact with each other in a dynamic way.

**Conclusions:**

Using a framework approach, these country case studies demonstrate common factors that influence how drug shops can strengthen health systems. These learnings can help inform the design and implementation of successful strategies to engage drug shops towards sustainable systems change.

## Background

Improving access to essential medicines has the potential to contribute to stronger, more equitable health systems, towards universal health coverage (UHC) and improved health outcomes [1–3]. Despite global attention directed towards essential medicines for decades, equitable access, affordability and appropriate use of essential medicines remains problematic, particularly in low and middle income countries (LMICs) [4–6]. The ongoing nature of the problem suggests that complex systems-level constraints, including those at the level of individuals and households, service delivery and policy underlie these issues. Therefore, looking at the issue through a systems lens to inform the design of programmes and policies to improve access is essential to overcome these challenges [7].

In several countries around the world, the private sector plays an important role in health systems, including in the direct provision of healthcare services and of medicines. Engaging the private sector may thus be important in efforts to move towards UHC [8, 9]. The private sector spans a whole range of providers. They include the for-profit formal private sector, that is often concentrated in urban areas and caters to better off populations, non-governmental and faith based providers, who are often the only sources of care in rural and hard to reach settings, as well as private medicine retailers or drug shops. Community pharmacists and private medicine retailers can play an essential role in the appropriate use of medicines, which can improve health outcomes [2].

Private medicine retailers, or drug shops, often supply a large portion of essential medicines, basic healthcare services and health advice in LMICs [10–12]. Literature shows in these settings, they are often individuals’ first and sometimes the only point of contact with the healthcare system [10, 13]. Their many advantages are well-studied. First, because of their number and presence in many communities, including rural and slum communities, they can help overcome geographic barriers to care [13, 14]. They frequently have long opening hours, providing more convenience to consumers and their perceived confidentiality and non-judgmental practices may encourage patronage from patients who fear stigma from other providers [12, 13, 15]. Because they service populations, where geographical and socioeconomic factors may otherwise limit access to care, they have the potential to contribute to stronger, more equitable health systems [11, 12, 14]. Through improving the affordability and accessibility of essential medicines and other basic healthcare, drug shops can, therefore, help progress towards UHC [16, 17].

Despite these many benefits, some drawbacks exist and these present opportunities to improve pharmacy practice [2]. Drug shops often sell medicines with little history-taking or counselling to clients and stock and dispense popular medicines at the expense of following treatment guidelines [11, 18]. They may stock poor-quality medicines, may not refer clients onwards when necessary, and have been noted to provide medicines and services beyond their legal scope of practice and that they are not trained to provide [12, 19]. Their unauthorized sale of antimicrobials can play a significant role in contributing to the threat of antimicrobial resistance [20, 21]. This is compounded by sub-optimal regulation and compliance with regulatory frameworks [22], which raises concerns about appropriate dispensing practices and quality of care, among other issues [14, 23].

Recognising the important contribution that drug shops can make in strengthening health systems through enabling access to medicines and health care among the poorest and most vulnerable groups of the population, several recent initiatives have been launched to engage drug shops to improve the quality of their services. These range from initiatives that focus on training, to policy and regulatory initiatives to collaborative partnerships between public and private sector providers [13, 15, 19, 24, 25].

While there is a significant body of literature around initiatives targeting drug shops and associated outcomes, much less is known about the specific processes and mechanisms that contribute to initiative success, and through which such initiatives strengthen health systems. Understanding these mechanisms is important to informing the design and implementation of these initiatives.

In their systematic review of eighteen initiatives targeting drug shops in LMICs, Smith et al. find that interventions focused on education can lead to improvements in quality of care, but the effects of education alone were probably short-lived and limited to specific outcomes, leading researchers to conclude that education alone is not able to achieve sustainable and broad improvements in practice [13]. Because this improvement in quality of care after training was seen across many initiatives in various settings, Smith et al. suggest that this is context independent. No other context independent determinants of success were highlighted by this study [13]. Kitutu et al. agree that education contributes to initiative success and add that supervisory visits and community sensitisation enable better compliance to guidelines [14]. Miller et al. also demonstrate that while knowledge is necessary, it is not sufficient in ensuring correct management by retail pharmacies. They further find that profit-maximising strategies are often linked to poor practices [18].

To better understand such initiatives and how these can contribute to stronger health systems, the Alliance for Health Policy and Systems Research, WHO in conjunction with the Implementing Best Practices Initiative at WHO and with support from USAID launched a programme of implementation research in early 2019. Research teams based in low-and-middle-income countries were eligible to apply, with projects completed in 2020. Details about the research programme, and a summary of each of the published articles brought together in this supplement, are provided in the introductory editorial.

This cross-national paper aims to explore the constellation of factors that influenced why certain initiatives were perceived to be successful, whereas others were less so. It also aims to synthesise key learnings from the country case studies supported under this research program to shed light on how drug shops could play a more effective role in stronger health systems. We hope that this information will aid national and sub-national decisionmakers and funders interested in integrating drug shops into health systems in the design, implementation and ongoing improvement of such initiatives for sustainable systems change.

### Framework

There are a number of frameworks that examine the various determinants of access to medicines [7, 26–28]. Bigdeli et propose a framework that embeds access to medicines within a health systems perspective, emphasising the dynamic and complex relationships between individual components [7]. This framework encourages consideration of country experience at five levels: at the level of the individual, household and community, at the level of health service delivery, at the level of the health sector, at the national level and finally at the international level. In this paper, we use a modified version of this framework to present the reflections and learnings from each of the countries studied at three levels most relevant to drug shops—the micro (level of the individual) the meso (health service delivery) and the macro (health sector and national context).

## Data sources and methods

Seven country case reports, as well as related papers included in this supplement, were prepared as part of the Alliance-supported research program Strengthening Health Systems: The Role of Drug Shops. These documents were the primary source of information for this review paper [29–42]. The countries included in this analysis were Bangladesh, Indonesia, Myanmar, Nigeria, Tanzania and Zambia. Case studies are summarized in Table 1.

**Table 1:** Description of country context and initiative

Country and Authors	Background and context	Aim of initiative	Description of initiative	Description of study
Bangladesh Nizame et al.	Drug shops are a major source of healthcare for poor and disadvantaged populations in Bangladesh. Antibiotic stewardship is a challenge associated with antimicrobial resistance. Unregulated antibiotic sales by drug shops, therefore, contributes to this problem	To prohibit the sale and distribution of antibiotics without a prescription	Government of Bangladesh developed a National Drug Policy to lay out guidelines for appropriate sale and dispensing of antibiotics	Research group explored attitudes towards this policy through qualitative interviews towards informing the development of social and behavioral communication change materials
Indonesia Ferdiana et al.	Indonesia has the second highest number of malaria cases in South East Asia. Private health providers are often the first port of call for antimalarials, due to access factors, but they are unregulated and do not necessarily comply with standardized treatment guidelines, potentially increasing resistance	To implement a public–private partnership to increase access to quality antimalarials for patients, and increase adherence to guidelines for pharmacies	District health office provides free antimalarials to drug shops, ensuring quality and stock of guideline-adherent therapy. Drug shops are permitted to dispense these only with prescription and laboratory confirmation of malaria	Research group analyzed secondary data on malaria cases and the use of antimalarials by drug shops, as well as conducted interviews and focus group discussions on the acceptability of the partnership among key stakeholders
Myanmar Thein et al.	TB and malaria are important causes of morbidity and mortality in Myanmar. Drug shops are the first or only port of call for many people, especially for those in urban slums, for their healthcare needs	To integrate drug sellers into the healthcare system, enabling them to provide TB and malaria screening, testing, case notification and referral according to national guidelines	PSI/Myanmar provided training on guidelines and appointed one field supervisor per township to support drug sellers and patients with these services	Research group conducted a mixed-method study involving a quantitative survey and qualitative interviews to understand the barriers to service provision by drug shops currently engaged in the program
Nigeria Oluwasanu et al.	Injectable contraception is a popular family planning choice for Nigerian women but can’t be legally provided by PPMVs. PPMVs are the primary source of contraception due to geographical, financial and sociocultural factors. PPMVs have been providing injectable contraception regardless to meet customer demand	Training of PPMVs to deliver injectable contraception and to regulate this currently unregulated market. Initiative was introduced as a pilot program	Training on injectable contraception, as well as monitoring and follow-up at 3, 6 and 9 months. This was accompanied by a time-bound policy change allowing PPMVs to provide injectable contraceptives for the duration of the pilot	Research group conducted a mixed-methods study involving quantitative questionnaires and qualitative interviews to elucidate the processes through which this intervention influenced women’s utilization of injectable contraception
Nigeria Uneke et al.	PPMVs are often the first and only point of care for family planning, treatment of childhood illnesses, antibiotic prescribing and malaria care. They remain somewhat unregulated	Given the role of PPMVs in providing healthcare to large sections of the population, the aim is to integrate and regulate their operations to help contribute to moving towards UHC	Implementation of National Drug Policy on appropriate family planning services, malaria care and antibiotic dispensing by PPMVs	Research group conducted a self-administered questionnaire for PPMV staff, nursing mothers and young people to understand PPMVs knowledge of National drug policy and subsequent behaviours, as well as nursing mothers and young people’s access to and views on these services
Tanzania Shekalaghe et al.	Many patients seek healthcare from Accredited Drug Dispensing Outlets (ADDOs). These outlets are part of a public–private partnership using an accreditation approach to improve access to quality medicines rurally. However, poor communication between ADDOs, community health workers and health facilities may be associated with inadequate referral and integration of care	To better integrate ADDOs into the system by increasing communication pathways and referral between these three groups	A training initiative to educate each of these groups on the roles and responsibilities of the others and informally convene ADDOs, Community health workers and health facilities	Research group used in-depth interviews to understand relationships between these three groups, as well as barriers and facilitators to their linkage
Zambia Zulu et al.	There is inadequate access to quality medicines in rural Zambia, and especially for family planning. Specific issues include lack of skilled personnel, limited adherence to treatment protocols and dispensing drugs without prescriptions	To better regulate drug shops to increase the quality of their service provision and to increase access and quality of medication for vulnerable populations	Guidelines on operating Health Shops developed by Zambian Medicines Regulatory authority (ZAMRA), outlining key standards such as personnel requirements, medication supply, and record keeping. Guideline training also conducted	Research group conducted documentary review and qualitative interviews to understand the current acceptability of these guidelines among key stakeholders

Using the sources above, each initiative was first summarized in terms of aims, description of activities and outcomes. Then, Bigdeli et al.’s theoretical framework was applied within a partially grounded approach to analyze each country case study [7, 43]. This involved extracting data under each element of the framework, as well as allowing new categories to emerge from the data. The framework was then modified slightly, so as to not force the data to fit the framework. A cross-case analytical approach was employed, specifically looking at agreements and variations between countries, to provide insights that may be generalizable across the cases studied. Data was then refined and lessons distilled. This approach is widely used for testing theories, as well as building new ones [44]. We aimed to identify the role of individual variables within the framework towards stronger health systems, as well any interactions between them. To address potential reflexivity, co-authors independently reviewed the themes and interpretations presented here.

No additional human subjects were contacted for the purpose of this analysis; therefore, independent ethics approval was not sought beyond the individual approvals obtained for each country case study.

## Findings

Table 1 summarizes the background and context of these initiatives, briefly describes the aims of each initiative and the research focus of each study. While some teams focused on improvements in knowledge, behaviours or health outcomes from the initiatives, all teams aimed to understand the facilitators and barriers to initiative success. Thus, they explored the processes and mechanisms behind why initiatives were successful, or otherwise.

As can be seen from Table 1, the range of initiatives examined was quite diverse, ranging from broad to specific, and from single component to multi-component. The country case studies below show that drug shops do not only increase access to essential medicines, but also health services, specifically in identifying TB cases, providing malaria and TB diagnostics and providing reproductive health services. Health advice, specifically around HIV counselling and treatment of coughs and colds, was also provided.

Table 2 summarizes reported outcomes of initiatives for each country case study. Where provided by country case studies, Table 2 presents indicators around each initiative’s intended outcome, enabling an assessment of the initiative’s performance or relative ‘success.’ Indicators vary between countries, making direct comparison of outcomes and success challenging.

**Table 2:** Reported outcomes of initiatives

Country and Authors	Description of initiative and study aims	Outcome measured	Method of measurement	Finding
*Service delivery initiatives*				
Indonesia Ferdiana et al.	Public Private partnership implemented for antimalarials in Indonesia. Research group analyzed secondary data on malaria cases and the use of antimalarials by drug shops, as well as conducted interviews and focus group discussions on the acceptability of the partnership among key stakeholders	Malaria cases specifically reported by drug shops pre and post intervention as a percentage of malaria cases reported in the district overall	Malaria case reporting data to district health information system	Increase from 6.9 to 30.7% in percentage of overall cases of malaria reported by drug shops
Myanmar Thein et al.	PSI/Myanmar provided training on guidelines and appointed one field supervisor per township to support drug sellers and patients with TB and malaria services. Research group aimed to understand provision of these services and any barriers drug shops currently engaged in the program	Percentage of drug sellers who used correct criteria for determining a client as a presumptive TB case	Quantitative questionnaire developed by PSI/Myanmar	86.4% Referring all symptomatic TB clients using the correct criteria TB
		Percentage of drug sellers using correct definition for suspected malaria cases and referring these according to guidelines		87.7% Using correct definition and referring according to guidelines
Nigeria Oluwasanu et al.	Training on injectable contraception, as well as monitoring and follow-up at 3, 6 and 9 months conducted in Nigeria. Research group aimed to elucidate the processes through which this intervention influenced women’s increased utilization of injectable contraception	Percentage of PMVs knowledge assessed as complete on injectable contraception after participation in training	Quantitative questionnaires of PMVs administered by research group	96% of PMVs for Depo Provera 86.4% of PMVs for Noristerat 83.6% of PMVs for Sayana press
		Percentage of PMVs that perceived training improved knowledge and skills in injectable contraceptive service provision		85.7% Perceived that training improved knowledge and skills
		Percentage of PMVs that perceived training improved knowledge and skills in family planning counselling		85% Perceived that training improved knowledge and skills
Tanzania Shekalaghe et al.	Training initiative to educate each of these groups on the roles and responsibilities of other groups, and informally convene ADDOs, Community health workers and health facilities in Tanzania. Research group aimed to understand relationships and referrals between these three groups, as well as barriers and facilitators to linkage	Whether CHWs refer manageable cases of under five children to ADDO shops	In-depth interviews	25% Pre-training, 95.8% post-training
		Whether CHWs felt to be appreciated by health facility staff		65% Pre-training, 91.7% post-training
		Qualitative assessment of relationships		Relationships did not exist pre-training, therefore, much improved relationships among the three groups post training
Nigeria Uneke et al.	National Drug Policy on family planning services, malaria care and antibiotic dispensing by PPMV was implemented in Nigeria. Research group aimed to understand PPMVs knowledge of this policy and subsequent behaviours, as well as nursing mothers and young people’s access to these services	Mean level of agreement by nursing mothers on various domains of accessibility (1 = Strongly disagree, 2 = disagree 3 = Unsure, 4 = agree 5 strongly agree)	Questionnaire for nursing mothers developed by research group	PPMVs are easily accessible to rural populations—4.2 Sale of medicines without prescription is rampant—3.8
		Mean level of agreement by youth on various domains regarding PPMVs (1 = Strongly disagree, 2 = disagree 3 = Unsure, 4 = agree 5 strongly agree)	Questionnaire for youth developed by research group	PPMV shop owners and operators generally have low health knowledge about proper treatment for common illnesses, such as malaria and diarrhoea and poor health treatment practices—3.1 Dispensing medicines without prescription should not be encouraged—4.0
*Policy initiatives*				
Zambia Zulu et al.	Guidelines on operating Health Shops developed by Zambian Medicines Regulatory authority (ZAMRA). Research group aimed to understand the current acceptability of these guidelines among key stakeholders	Qualitative assessment of acceptability of guidelines	In depth interviews	*Facilitators to acceptability:* Comprehensive training on guidelines High level buy-in among stakeholders *Barriers to acceptability:* High cost of implementation Limited infrastructure and capacity to upgrade current shop to fit guidelines
Bangladesh Nizame et al.	Government of Bangladesh developed a National Drug Policy to lay out guidelines for appropriate sale and dispensing of antibiotics. Research group explored knowledge of and attitudes towards this policy	Knowledge of national drug policy and barriers to compliance with appropriate antibiotic stewardship as laid out by this policy	Qualitative assessment from key informant interviews and co-design workshops with drug shop operators	Low awareness of policy among drug shop owners *Barriers:* Drug shops demanded right to prescribe antibiotics Lack of qualified physicians available to prescribe antibiotics Customer demand for antibiotics

Table 3 describes specific factors that, either by their presence or absence in certain settings, positively or negatively influenced the overall success of initiatives targeting drug shops, organised under micro, meso and macro level factors. These factors are presented in a disaggregated way here for ease of analysis. However, factors shape—and are shaped by—each other and have non-linear effects on health systems.

**Table 3:** Factors contributing to success or failure of initiatives

Factor	Towards or against successful outcome?	Countries in which it was present and potentially influenced outcome	Countries in which factor was absent/suboptimal and lack of factor influenced outcome
*The micro level—individuals, households and communities*			
Communities choose drug shops	Towards success	Indonesia Nigeria (Oluwasanu) Nigeria (Uneke) Bangladesh Myanmar Zambia Tanzania	
Positive relationship between drug seller and client	Towards success	Nigeria (Oluwasanu) Myanmar^1^	Myanmar^1^ Nigeria (Uneke) Zambia Bangladesh
*The meso level—health service delivery and resources*			
Client pressure	Against success	Indonesia Nigeria (Oluwasanu) Nigeria (Uneke) Bangladesh Myanmar Zambia Tanzania	
Training	Towards success	Myanmar Nigeria (Oluwasanu) Nigeria (Uneke) Tanzania Zambia	Bangladesh Indonesia
Procurement challenges	Against success	Nigeria (Oluwasanu) Indonesia Myanmar	
Financial and administrative burden	Against success	Zambia Indonesia Myanmar	
Positive drug shop owner attitudes towards initiative	Towards success	Indonesia Zambia Nigeria (Oluwasanu)	Bangladesh
*The macro level—health sector and national context*			
Supervision, monitoring and regulation	Towards success	Nigeria (Uneke) Zambia^2^ Myanmar	Indonesia Bangladesh Zambia^2^ Nigeria (Oluwasanu) Tanzania
Multi-stakeholder collaboration and high-level buy-in	Towards success	Nigeria (Oluwasanu)^3^ Bangladesh Zambia Tanzania Myanmar Indonesia	Nigeria (Oluwasanu)^3^ Indonesia Tanzania

^1^The presence of Myanmar in both columns should be interpreted to mean that a positive relationship between drug seller and client was noted to positively affect outcomes, and a negative relationship between drug seller and client was noted to negatively affect outcomes

^2^In Zambia, while supervision was noted to be helpful, regulation was inadequate which negatively influenced the ability of the initiative to contribute to stronger health systems

^3^In Nigeria, multi-stakeholder collaboration and buy in was noted to be helpful, but there was one stakeholder group creating opposition to the policy change, which posed a barrier

The remainder of the findings section presents key factors at the micro, meso and macro level, under a modified version of Bigdeli’s Access to Medicines framework.

### The micro level—individuals, households and communities

Individuals, households and communities, as end-users of drug shops, are key when considering the success of initiatives. Micro level factors that influence the ability of drug shops to contribute to stronger health systems include community demand for drug shops as well as relationships between them and the communities they serve.

#### Communities choose drug shops

All seven country case studies presented here agree that individuals, households and communities choose drug shops as their first, and sometimes only, point of contact with the healthcare system. There are many reasons for this. First, their proximity to clients, especially in suburban and rural areas, where there are limited formal health facilities for patients. Second, they lower financial barriers to accessing essential medicines and basic health services. Third, for convenience, in that drug shops offer longer operating hours and an alternative to the long waiting times often seen in public facilities. These factors are not only applicable to essential medicines, but also for service delivery due to their serving as points of care for specific health services, such as TB, Malaria and contraceptive services.

#### Relationship between drug shops and clients

In many country case studies, a good pre-existing relationship between drug shops and their clients facilitated drug shops’ ability to provide medicines and services. For example, in Nigeria, PPMVs were based in the community and already had a rapport with households. Clients preferred them due to their friendly and non-judgemental nature. 99.2% of injectable contraceptive users went back to the same PPMV regularly. Similar factors were noted to be important in Zambia and Myanmar.

One barrier to the engagement with drug shops at the individual level is trust; a lower level of community trust may result in lower utilisation of services and medications. In Myanmar, it was reported that clients had a lack of trust in drug sellers, as they are not medical doctors or medically trained personnel. Thus, it was sometimes difficult for them to convince clients that they needed a referral. Similarly, low levels of community trust in drug shops due to their lack of formal education were noted in Nigeria. Initiatives that specifically target improving quality by increasing training and ensuring medication quality may help address these issues.

### The meso level—health service delivery and resources

Meso level factors operate at the level of the health service delivery system. Considering meso-level factors is important in understanding the mechanisms by which drug shops can play a role in health system strengthening. Factors influencing initiative success at this level included client pressure, procurement challenges, financial and administrative factors associated with the initiatives, as well as the importance of having sufficient, well trained human resources for health.

#### Client pressure

In all case studies, community demand is a key driver of drug shops’ willingness to supply unregulated medications. As drug shops are private providers who primarily operate as businesses, they are influenced by client pressure and profit motivations. For example, in Nigeria, in relation to antibiotics, PPMVs were reported to have high knowledge on national guidelines and the penalties associated with inappropriate dispensing. However, many PPMVs dispensed antibiotics without a prescription, despite this being prohibited, with resulting consequences including possible imprisonment. One explanation for this was that.

*‘refusing to dispense certain medicines/drugs without prescription will negatively affect…sales and profit* [37]*’*

Drug shop owners also provided prohibited services and medications as reported by the Bangladesh and Zambia studies due to customer demand. In Indonesia, community pharmacies admitted lying to patients who came in requesting antimalarials without prescription, preferring to state that the medicine was out of stock rather than angering potential future customers.

We observe that such pressure arises partially from community misinformation. For example, in Bangladesh, it was reported that clients refuse to buy a complete course of antibiotics, as they feel ‘cured’ after a partial course. Similar behaviours were noted in Myanmar and Nigeria. This decreases the success of initiatives targeting drug shops, especially interventions encouraging drug shops to adhere to specific dispensing practises or guidelines.

#### Procurement challenges

Initiatives had different procurement strategies. In Nigeria, drug shops were reported to be connected with reliable commercial suppliers, and in Indonesia, they were directly provided with high-quality medications by the district health office. Both strategies worked well when medications were easy to source. However, when their primary supplier was out of stock of medications, drug shops sourced them from the open market to satisfy customer demand. This was costly and may have compromised quality. Findings from Zambia highlight that a lack of reliable supply affected affordability of medications. Thus, an absence of reliable procurement sources impacts on medicine quality and price.

#### Financial and administrative burden

Implementation of initiatives was hampered by the financial and administrative burdens they sometimes imposed. For example, in Zambia, the guidelines around the initiative required putting in place new infrastructure for drug shops, which was very costly for two reasons: they needed initial capital and had to close for a period to complete renovations.

Accreditation, registration, and other fees were also reported to be burdensome. In some settings, fees had to be paid in the capital city, with associated time, workforce and travel costs. In Indonesia, antimalarials were provided at no cost to the drug shops, but the administrative obligations created by procurement processes were a cumbersome barrier to an already stretched health workforce. In Myanmar, drug shops reported a lack of time to follow-up patients as per the guidelines. In many settings, drug shop owners are not incentivised specifically for these initiatives. Consequently, many drug shops in Zambia and Indonesia opted out of participating in initiatives, as burdens outweighed benefits. If the time and workforce costs associated with extra administrative requirements for compliance with initiatives are not addressed or compensated for, these initiatives are less likely to succeed.

#### Health workforce

Studies from Bangladesh, Indonesia, Nigeria, Zambia and Myanmar show that the lack of qualified physicians or heath personnel, especially in rural areas, can be partially addressed by drug shops, with adequate training and monitoring. For example, in Tanzania, there are currently over 27,000 trained dispensers, equating to approximately 48 personnel per 100,000 population [42]. Recruiting and upskilling drug shop personnel to dispense a limited list of medications was a strategy noted in Bangladesh, Indonesia, Nigeria and Zambia.

A common theme that emerged from the data was that high quality training positively influences both attitudes and knowledge of drug shop personnel. For example, in Nigeria, PPMVs attended a 5 day training on family planning counselling, sale, referral and administration, which was reported to be a key causal mechanism for the resultant increased uptake of injectable contraceptives. The proposed mechanism of this was that training improves PPMVs knowledge, skills and motivation to provide injectable contraceptive services. One PPMV participant stated that:

*‘after the training there is now no more doubts and fear *[32]*’*

PPMVs reported increased confidence in their abilities after the training. Similarly, training sessions run in Zambia and Tanzania for drug shop staff resulted in increased knowledge, which may help drug shops in correctly diagnosing and consequently dispensing evidence-informed treatments. This may potentially increase service quality.

In some contexts, a lack of training was noted to be problematic. In Bangladesh, one drug shop owner stated that.

*‘we want to learn the proper use of antibiotics and we will carry our learning to the people. Government’s responsibility is to train us through various conferences, seminars and training*[29]*’*

While this shows a demand for training, in some contexts, coverage of training was inadequate.

A lack of training also influences community trust. In Nigeria, 60% of youth interviewed stated that a barrier to their use of PPMVs is a lack of training and education of PPMVs.

Country case studies illustrate tangible strategies for high-quality training. For example, training in Nigeria included using a context-specific curriculum, taught through workshops with both didactic and practical sessions. Graduation and certification depended on meeting certain competency thresholds. In addition, ensuring that the trainers themselves were highly educated and disciplined was helpful.

However, specific shortfalls for training still remained. In some settings, particular areas of in-depth knowledge were lacking. In addition, knowledge was not always retained after training. In Nigeria, while knowledge increased in post-training assessment in 47 out of 50 indicators, at 9 months follow-up, knowledge had been retained in only 29 indicators. Solutions to these were tailored to context, for example, in Nigeria, an implemented strategy to mitigate this was the development of job aids with specific in-depth information which drug sellers could reference. When used, these increased frontline worker adherence to policies and guidelines.

#### Positive drug shop owner attitudes towards initiative

When drug shop staff held positive views towards initiatives, this seemed to shape their implementation in positive ways. In Indonesia, most participants expressed positive attitudes towards the partnership. There was a social motivation to contribute to malaria elimination, as malaria is a significant public health problem in the district. Similar public health motivation was noted in Zambia. However, in Bangladesh, despite training, drug shops did not adhere to antimicrobial stewardship principles, as staff believed they had the expertise and right to prescribe antibiotics. This shows that if drug shops harbour negative attitudes towards initiatives, this hampers implementation.

### The macro level—health sector and national context

Macro-level factors operate at the level of the programme and policy, as well as national context. Macro-level and contextual factors can be important determinants of initiative success. These included supervision, monitoring and regulation of initiatives, collaboration between various stakeholders and high-level buy-in.

#### Supervision, monitoring and regulation

Supervisory support was important to the success of many initiatives. In Myanmar, a specific staff member in each township was allocated to support drug shops and their clients. Their supervisory role was essential to ensuring compliance with guidelines. In addition, in Myanmar, results collected from a routine app-based assessment tool were used to determine the frequency of supervision visits and tailor feedback. In Nigeria and Zambia, support and supervision activities so drug shops could communicate their challenges and seek advice were helpful. Conversely, in Indonesia, there was no regular supervision. This led to waning motivation among drug shops.

Framing and intention of visits as supervision and support versus regulation and compliance was also important. In Nigeria, some drug shops experienced harassment from regulators, an experience that was echoed in Zambia. In addition, one PPMV vendor from Nigeria remarks that.

*‘the major problem is that the government drugs/medicines regulatory agencies are not doing enough in the monitoring of the operations of PPMVs to ensure strict adherence to the national drug policy *[38]*’*

They argue that drug laws and policies are adequate, falling short only in their implementation due to corruption, communication gaps, lack of adequate funds and lack of vehicles. Similarly, in Bangladesh, a planned monitoring and regulation system was never implemented due to resource shortages, so there was no clear mechanism for ensuring compliance.

Regulatory visits that were concerned with enforcing guidelines and issuing sanctions were overall less helpful than supervisory visits to identify implementation issues and advise on their correction. In some initiatives, a shift in mindset was required among certain stakeholders who may have never previously worked with drug shops, such as public sector actors and regulators.

#### Multi-stakeholder collaboration and a favourable policy environment

Another factor contributing to initiative success was collaboration between multiple health system levels. In Nigeria, multi-stakeholder collaboration facilitated a favourable policy and legislative environment, with buy-in from federal and state ministries of health, the department of pharmaceutical services, and the national reproductive technical working group allowing PPMVs to provide injectable contraceptives on a temporary basis to facilitate a pilot programme. There was also an institutional training and support mechanism for drug shops, which led to increased credibility and community acceptance. Partnerships between drug shops and the public sector had another collateral benefit. The increased interactions led to increased trust and better working relationships, resulting in increased referrals in Nigeria, Zambia, Myanmar and Tanzania.

Multi-stakeholder collaboration is a complex process as stakeholders have differing interests, values and power. In Indonesia, there were communication challenges between drug shops and the public sector and private physicians. Similarly, tensions between stakeholders in Nigeria negatively impacted initiative performance.

## Discussion and recommendations

Reflecting on the experiences of these case studies across six countries, we learn that there are many factors that may help initiatives targeting drug shops successfully achieve their intended outcomes. At the micro level, these factors are community demand for drug shops and a positive relationship between drug shops and their clients. At the meso level, facilitators of initiative success include training and positive attitudes from drug shops towards the initiative. Barriers include client pressure, procurement challenges and financial and administrative costs associated with initiatives. At the macro level, collaboration, high-level buy in and supervision, monitoring and regulation, all may influence whether initiatives are successful. Our findings agree with previous literature that at the individual level, drug shops have the potential to contribute to strong health systems through overcoming geographic, financial and administrative barriers to access [11, 13, 14, 23].

While single factors affecting success of drug shops are identified in this paper for simplicity of analysis and presentation, they are, in fact, part of a complex and dynamic health system.

In the cases studied, these factors did not have a linear effect on health systems. There are two additional characteristics of these factors to consider; first, the degree of contribution of each factor and second, the interaction among factors and between other aspects of the health system.

We observe that in different contexts, some factors were a stronger contributor than in others. For example, in Myanmar, supervisory support was noted to be a very strong contributor to initiative success. In Nigeria, safe injectable contraceptive provision was facilitated by high-quality, rigorous training. In Bangladesh, a major barrier to success was drug shop owners’ attitudes and held beliefs towards the initiative. In Zambia, implementation struggled due to a significant financial and administrative burden associated with the initiative. From this, we can learn that factors affecting success are context-specific. We, therefore, recommend that initiatives should be tailored to context.

Also, factors are interrelated and can re-enforce and balance each other. In general, we can see that the more factors the contributing to initiative success existed, the more successful the initiative was, and vice versa. However, a causal or linear relationship for specific factors cannot be drawn here. Nevertheless, when we examine the relatively more successful case studies of Myanmar and Nigeria, we observe common facilitators of success included customer demand for drug shops, trust and a good rapport between drug seller and client, high-quality training, and harmonious multi-stakeholder collaboration.

Our findings agree with previous literature that education is a context-independent determinant of initiative success and that recognises the important role that training plays in strengthening the health workforce [13, 15, 45]. Training the health workforce for safe provision of primary healthcare may improve coverage of essential health services and accelerate progress towards UHC. Training may also lend legitimacy to drug shops for communities. It counteracts fears of suboptimal education for drug shop staff and leads to increased community trust in knowledge and skills, possibly increasing demand for drug shops. It also increases their legitimacy in the eyes of health system stakeholders, such as community health workers and other health system officials. It may help change drug seller attitudes towards initiatives, enhancing their own legitimacy and empowering them to address public health problems. This is one example of the synergy between factors.

Specific aspects of training found to be helpful were practical, skill-based sessions, a curriculum adapted to context, assessments, thresholds for certification and highly educated trainers themselves. However, in some settings, specific areas of in-depth knowledge were lacking, and knowledge was not completely retained at follow-up. In Nigeria, a strategy implemented to mitigate this was the development of job aids with specific in-depth information which drug sellers could reference. When used, these increased frontline worker adherence to policies and guidelines.

Stakeholder engagement and high-level buy in and communication was found to be essential for a successful initiative. However, stakeholders may have competing interests, so jointly determined goals may help build long term relationships. Brinkerhoff asserts that key factors in building long term relationships include jointly determined goals, non-hierarchical structures and processes, collaborative decision making and synergistic interactions among partners [46]. High-level buy-in also increased community trust in drug shops: when health officials were visibly seen to be visiting and supporting drug shops in Nigeria, communities found them to be more credible. This is another example of individual factors having synergistic benefits.

Both these successful settings faced procurement challenges. This may lead us to believe that these three factors—training, relationship between drug seller and client, and multi-stakeholder collaboration—are among the strongest constellation of facilitators of initiative success. This may also lead us to believe that procurement challenges can be overcome when initiatives are otherwise strong.

In relatively less successful cases, such as Zambia and Bangladesh, national drug policies were well-designed, but there were significant implementation challenges. In these contexts, despite multi-stakeholder collaboration and high-level buy-in during the design phase of the policy, common factors hampering optimal implementation included a lack of community trust in drug sellers, customer demand and drug seller willingness to supply unregulated services, and poor supervision, monitoring and regulation. We agree that ‘top-down’ initiatives exemplified in these national policies may be interpreted by drug sellers as impractical or undesirable [47]. Thus, ‘bottom-up’ approaches that address problems that drug shops and communities see as important and also take into account the need to build community trust may encounter less resistance [14].

Kitutu et al. provide an example of an initiative that increased legitimacy and trust in drug shops; this multipronged initiative included upskilling drug shops, establishing reliable supply chains and providing community education and awareness-building [14]. Another strategy towards addressing this was studied by Hutchinson et al., who found that the presence of malaria diagnostics and other visibly medicalised items—considered the domain of trained health workers—built confidence in drug shop abilities and enhanced legitimacy among communities [48].

Financial and administrative costs discourage drug shops from participating in initiatives. Specific strategies to address these issues in rural drug shops suggested by country researchers include streamlining procurement processes, allowing fees to be paid digitally or via phone and reducing or waiving fees to incentivise people to practice rurally, tackling issues of health system inequity. If financial incentives cannot be offered to drug shops, Indonesia’s case study suggests that non-financial incentives, such as capacity building workshops, or in the case of Nigeria, a formal certification to be displayed in the drug shop, be offered instead.

Poor policy implementation, due to inadequate capacity and an adversarial attitude from regulators causing fear, is another reason for poor intervention adherence that has been noted previously [11, 47, 49]. We find that supervision and monitoring visits are helpful for health workers and agree that to be effective, health workers need to be well-supported and well-resourced [50]. We suggest that such visits should be conducted on a regular basis to help identify implementation issues and advise on their correction.

Finally, we notice that the aim of the initiative and what disease(s) it targets may influence success. For example, drug shop owners were more enthusiastic towards malaria and TB initiatives compared to antimicrobial stewardship. There also seemed to be much more multi-stakeholder collaboration around these ‘immediate’ public health problems. This may be because antimicrobial resistance is perhaps too removed from day-to-day experience and is not immediately visible. In addition, a trusting, non-judgemental relationship between drug shops and their clients is particularly critical for family planning initiatives, where stigma remains a significant barrier to contraceptive use. Thus, what the initiative targets influences attitudes towards it.

**Table:** 

Key lessons and recommendations
Training of drug shop staff increases knowledge, skills and compliance to guidelines, as well as their credibility, thus improving community trust. There is a need to ensure high quality, continuous and affordable supply of essential medicines to enable drug shops to contribute to health systems strengthening efforts.
Supervision and monitoring visits should be conducted on a regular basis to help identify implementation issues and advise on their correction.
Multi-stakeholder collaboration and communication is essential. Stakeholders may have competing interests, so jointly determined goals may help build long term relationships. It can also help build policy and legislative environments that facilitate the role of drug shops in health systems strengthening.
Drug shop owners are motivated by customer demand and profitability of their shops. Financial and administrative costs discourage drug shops from participating in initiatives. They may need to be incentivised, either financially or otherwise, to participate.

**Fig. 1:**
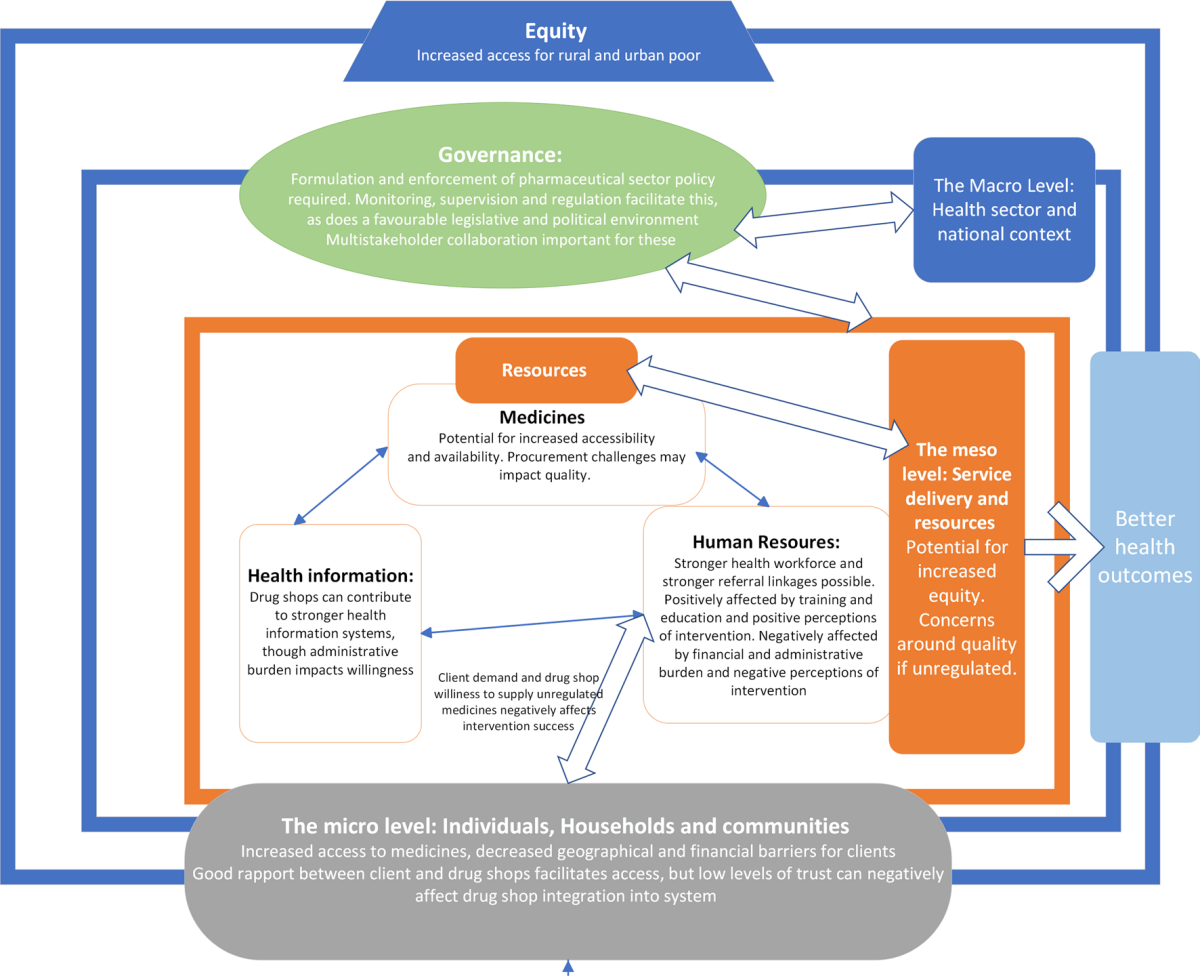
Drug shops through a health systems lens

With these project experiences, we populate a modified version of Bigdeli et al.’s framework (Fig. 1).

This figure shows the micro, meso and macro levels of the health system. It illustrates the key role that individuals, households and communities play in demanding medicines and services from drug shops and the equally important role of health resources and health services in relation to drug shops’ ability to supply them. It also situates drug shops within the macro-level context of a national health system, with associated governance and oversight influences. The overarching principle of equity is emphasised as a critical factor when designing and implementing initiatives for drug shops in health systems strengthening efforts.

There are some limitations of this study. First, each country case study measured initiative outcomes in different ways. Data collected across studies is, therefore, difficult to directly compare. Having similar projects and protocols implemented in all settings may have enhanced our ability to meaningfully compare between countries, and potentially enhanced the generalisability of our findings. Second, while some outcomes were measured over various timepoints, there is very little longitudinal data in the country case studies. It is, therefore, difficult to determine how various factors impact domains of the health system over time. Third, these six case studies only cover a segment of LMICs. While Miller et al. show that pharmacy performance in Asia is relatively consistent across countries and over time [18], LMICs are not a homogenous group and there are large variations between their health systems and pharmaceutical practices [2]. Health care needs may be more acute and resources may be more stretched in low-income counties when compared to upper-middle-income settings [13]. For example, the important recommendation to ensure some trained pharmacy workers are in place may be more feasible from a resourcing perspective in the latter. Other important contextual factors to consider include the difference between rural and urban settings, capacity for regulation and the existence of a national public health program targeting a specific disease [13, 18]. Therefore, these findings should be applied with caution to other settings.

Despite these limitations, we believe that by comparing country case studies, these findings help understand the factors that influence why initiatives are successful and how they potentially contribute to health systems strengthening, and provide tangible recommendations for the design and implementation of initiatives related to drug shops in LMICs.

## Data Availability

The datasets analysed during the current study are available from the corresponding author on reasonable request.
